# Comparative analyses reveal potential uses of *Brachypodium distachyon* as a model for cold stress responses in temperate grasses

**DOI:** 10.1186/1471-2229-12-65

**Published:** 2012-05-08

**Authors:** Chuan Li, Heidi Rudi, Eric J Stockinger, Hongmei Cheng, Moju Cao, Samuel E Fox, Todd C Mockler, Bjørge Westereng, Siri Fjellheim, Odd Arne Rognli, Simen R Sandve

**Affiliations:** 1 Maize Research Institute, Sichuan Agricultural University, Sichuan, China; 2 Department of Plant and Environmental Sciences, Norwegian University of Life Sciences, ÅS, Norway; 3 Department of Horticulture and Crop Science, The Ohio State University/OARDC, Wooster, OH, 44691, USA; 4 Biotechnology Research Institute, Chinese Academy of Agricultural Sciences, Beijing, 100081, China; 5 Department of Botany and Plant Pathology and Center for Genome Research and Biocomputing, Oregon State University, Corvallis, OR, USA; 6 Donald Danforth Plant Science Center, Saint Louis, MO, 63132, USA; 7 Department of Chemistry, Biotechnology and Food Science, Norwegian University of Life Sciences, Ås, Norway

**Keywords:** *Brachypodium distachyon*, Cold climate adaptation, Ice recrystallization inhibition protein, Gene expression, Fructosyltransferase, C-repeat binding factor, Gene family evolution

## Abstract

**Background:**

Little is known about the potential of *Brachypodium distachyon* as a model for low temperature stress responses in Pooideae. The ice recrystallization inhibition protein (IRIP) genes, fructosyltransferase (FST) genes, and many C-repeat binding factor (CBF) genes are Pooideae specific and important in low temperature responses. Here we used comparative analyses to study conservation and evolution of these gene families in *B. distachyon* to better understand its potential as a model species for agriculturally important temperate grasses.

**Results:**

*Brachypodium distachyon* contains cold responsive IRIP genes which have evolved through *Brachypodium* specific gene family expansions. A large cold responsive CBF3 subfamily was identified in *B. distachyon*, while CBF4 homologs are absent from the genome. No *B. distachyon* FST gene homologs encode typical core Pooideae FST-motifs and low temperature induced fructan accumulation was dramatically different in *B. distachyon* compared to core Pooideae species.

**Conclusions:**

We conclude that *B. distachyon* can serve as an interesting model for specific molecular mechanisms involved in low temperature responses in core Pooideae species. However, the evolutionary history of key genes involved in low temperature responses has been different in *Brachypodium* and core Pooideae species. These differences limit the use of *B. distachyon* as a model for holistic studies relevant for agricultural core Pooideae species.

## Background

*Brachypodium distachyon* became the first Pooideae grass species to have its genome fully sequenced [1]. The *Brachypodium* genus is a phylogenetic sister group to the Triticeae (cereals) and Poeae (forage grasses) tribes, which provided compelling rationale for sequencing the *B. distachyon* genome to develop a model more suitable for temperate grasses than rice (*Oryza sativa*). *B. distachyon* possesses features typical of a model plant [2]; it is of relatively short height (15–20 cm), there are inbred lines with an annual and rapid life cycle (eight to twelve weeks), it’s genome is one of the smallest among diploid grass genomes (about 300 Mbp) [1], and it can be genetically transformed via *Agrobacterium-*mediated transformation [3]. Different ecotypes exhibit a range of adaptations to environments which also are important challenges faced in agricultural production systems, for example differences in flowering time, vernalization requirements [4], and disease resistance [5]. Altogether, these features make *B. distachyon* a suitable model plant for studying agronomic traits in Pooideae grasses.

**Figure 1:**
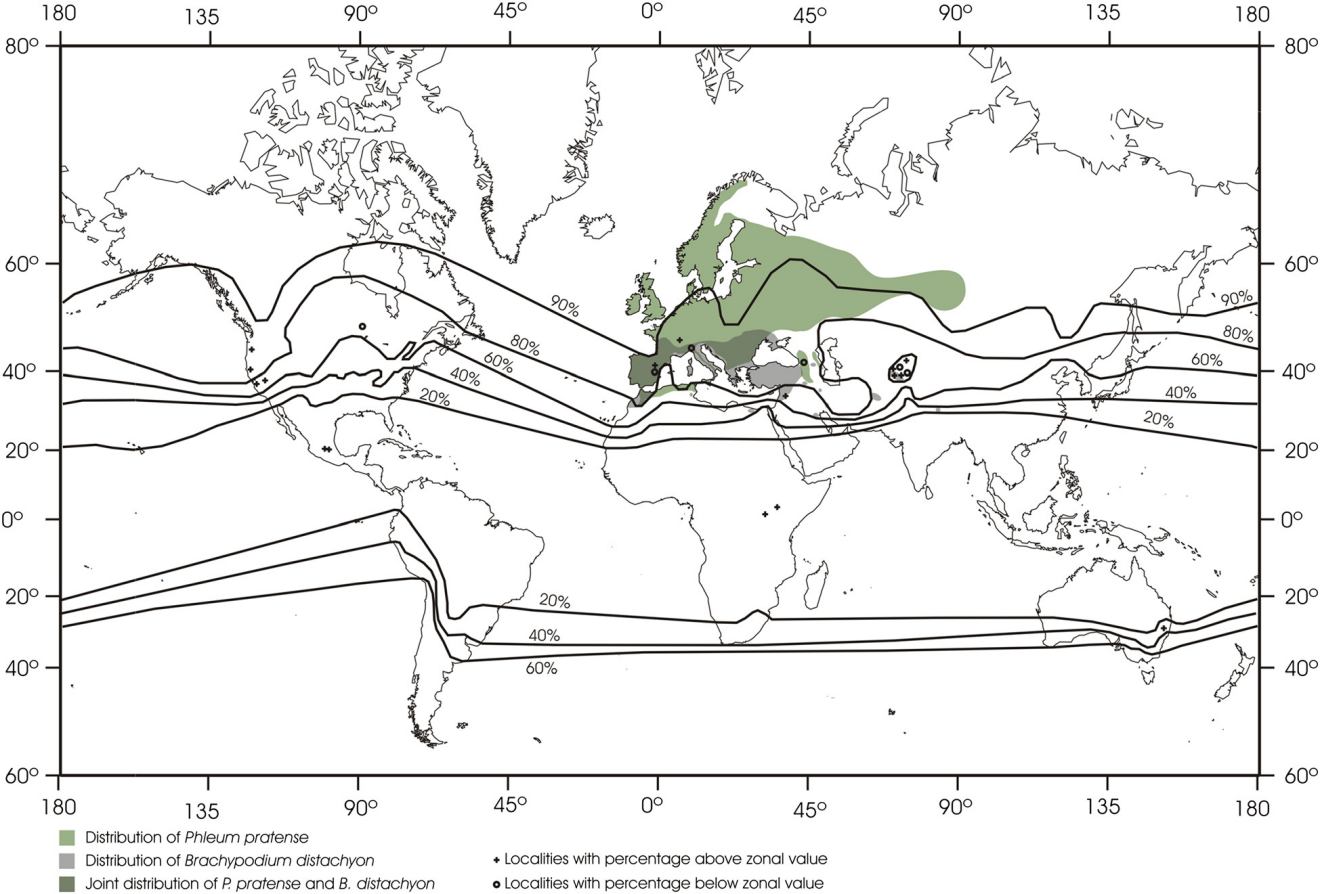
**Worldwide distribution of members of Pooideae species as percentage of the total grass flora.** The native distributions of *Brachypodium distachyon* and a typical cool season Pooideae forage grass, *Phleum pratense*, are indicated in two different green shadings. Data for distribution is taken from Hultèn and Fries [11] for *P. pratense*, and Flora Europaea [12], Filiz et al. [13] and data retrieved from the Global Biodiversity Data Portal (data.gbif.org, 2011-11-08) for *B. distachyon*. This figure (without *B. distachyon* and *P. pratense* distributions) was adapted from Hartley [6] with permission from CSIRO publishing. Pooideae is cited as Festucoideae in the Hartley [6] original paper.

A characteristic feature of species in the Pooideae sub-family is their adaptation to temperate ecosystems, which is reflected in the global distribution of Pooideae grasses [6] (Figure 1). The most recent common ancestor of Pooideae grasses was adapted to tropical or sub-tropical climates [7,8]. Subsequent radiation of Pooideae into cooler environments is thought to be associated with evolution of mechanisms involved in low temperature stress [9]. Hence, the adaptation of the Pooideae to cooler climates makes this group an ideal model system for studying adaptive evolution in plants [10]. Nonetheless, large intraspecific variation in tolerance to cold and freezing stress exists within Pooideae. Some Pooideae species (e.g. *Phleum pratense*) can tolerate extreme winter climates and has a species range which includes sub-arctic regions (Figure 1), while *B. distachyon* on the other hand is not adapted to extreme winter climates, which is reflected by the middle-eastern and Mediterranean geographical distribution (Figure 1).

Phylogenetic studies suggest that *B. distachyon* diverged from the core Pooideae approximately 35 million years ago [2] while key Pooideae-specific adaptations to cold climates evolved during the Eocene-Oligocene cooling period (34–26 Mya), after the *B. distachyon*-core Pooideae split [9]. If this is correct, shared ancestral molecular mechanisms involved in cold and freezing stress might differ between *B. distachyon* and agriculturally important species of the Triticeae and Poeae tribes (referred to hereafter as core Pooideae). 

Three Pooideae-specific genetic features involved in low temperature stress tolerance have been well described; (1) ice-recrystallization inhibition proteins (IRIPs), (2) fructosyltransferase genes (FSTs), and (3) expansions within the C-repeat binding factors (CBF) family. IRIPs are thought to minimize cell damage during ice formation by restricting ice crystal growth in the apoplastic space [14-16], a process known as ice crystal recrystallization. The ancestral IRIP gene in Pooideae is thought to have evolved from an LLR-protein kinase [17] by gaining an ice binding domain [16] through an expansion of a repeat motif (NxVxG/NxVxxG) [18]. In core Pooideae species extensive gene duplications have given rise to large IRIP gene families [18,19]. FSTs convert sucrose molecules into fructan sugars [20,21] and low temperature stress induces FST gene expression and fructan accumulation in core Pooideae species [22-24]. Both the introduction of FSTs into plants lacking endogenous FST pathways and over-expression of endogenous FSTs in core Pooideae species has shown to increase freezing tolerance [25,26]. CBFs are transcription factors that regulate suites of genes during drought and low temperature stress [27-29]. Two CBF subfamilies, CBF3 and CBF4, have undergone lineage specific duplications in the Pooideae [30] and the members of these CBF3/4-subfamilies are thought to play roles in Pooideae freezing stress [31-33].

Even though IRIP, FST, and Pooideae-specific CBF3/4 genes have been studied in great detail in core Pooideae species, a systematic study of homologs of these genes in *B. distachyon* is lacking. In this study we ask the question; to what extent are IRIP, FST, and Pooideae specific CBF genes conserved between *B. distachyon* and agriculturally important core Pooideae species? We answer this question by employing a suite of methods including comparative genomics, gene expression analyses, and characterization of carbohydrate metabolism. Our aims were to (1) assess the use of *B. distachyon* as a model to study mechanisms of low temperature stress responses in core Pooideae species, and (2) improve our understanding of the evolution of cold stress response in the Pooideae lineage.

## Methods

### Plant material, growth conditions and tissue sampling for gene expression studies

Four diploid inbred *B. distachyon* lines, Bd3-1, Bd21-1, Bd1-1, and Bd29-1 were used to characterize cold induced IRIP gene expression by quantitative real-time polymerase chain reaction (qRT-PCR). The seeds were kindly provided by Dr. David Garvin, University of Minnesota, USA. Bd3-1 and Bd21-1 originate from Iraq and are spring genotypes that do not require vernalization to induce flowering. Bd1-1 and Bd29-1 originate from northern Turkey and the Ukraine, respectively, and are winter genotypes which require long vernalization periods (six and 12 weeks respectively) to flower ( http://www.ars.usda.gov/SP2UserFiles/person/1931/GarvinLabCoreBrachypodiumdistachyonLineSet(2).pdf) [34,35]. For the microarray gene expression experiments only Bd21-1 was used.

In the qRT-PCR experiment 7 weeks old plants were used which had been established from seeds using the following growth conditions: 20/16°C day/night temperature and 16 h photoperiod with a photon flux density of 150 μmol m^-2^ s^-1^. Half of the plants were cold acclimated (CA) following the procedure outlined in Alm et al. [36] except that pre-acclimation was done at 12 h photoperiod, 12/6°C day/night temperature for 1 week. Plants were kept in CA conditions at 14 h photoperiod at a constant temperature of 1°C. Leaf tissues were collected from control plants (non-acclimated, NA) at the start of the pre-acclimation period and from CA plants at 4 h, 1 day and 10 days after the start of the cold acclimation period. To avoid experimental bias introduced by diurnal clock regulation of gene expression all samples were collected at the same hour of the day (in the morning) for each time point. Tissue for RNA extraction was sampled from different plants of each genotype at each time point. All sampled leaf tissues were frozen immediately in liquid nitrogen and stored at −80°C.

For the microarray experiment plants were grown in 16 hours photoperiod in a controlled growth room. The temperature was 23°C and the photon flux density was 200 μmol m^-2^ s^-1^. Cold experiments were conducted on three-week-old plants in a walk-in cold room at 4°C with a photon flux density of 200 μmol m^-2^ s^-1^. Control plants remained in the environmentally controlled growth room at 23°C. Experimental treatment began two hours post-dawn (10:00 am). Leaves and stems (total above ground tissues) from individual plants were collected at 1, 2, 5, 10, and 24 hours after experiments were initiated.

### Identification of *Brachypodium distachyon* IRIP-homologs and design of paralog specific primers

*Brachypodium distachyon* IRIP homologs were identified through web-based blast search ( http://www.Brachypodium.org) using IRIP genes from *Lolium perenne* (AY968588; EU680848; EU680850; EU680851) as queries. Multiple alignments of translated IRIP genes were made with default settings on the MAFFT web server [37] to verify that *B. distachyon* IRIP genes contained the typical NxVxG/NxVxxG-repeat ice binding domain. IRIP-paralog specific primers were designed using primer3 [38] such that there were mismatches between IRIP-paralogs in the 5’ end of at least one primer for each primer-pair. IRIP paralog specificity was verified by cloning the PCR-product of the paralog specific primers pairs using a TOPO TA cloning kit (Invitrogen) and subsequent sequencing of five to ten clones. Final TaqMan MGB probes and primer sets for the quantitative reverse transcriptase PCR analyses (qRT-PCR) of IRIP genes were designed using Primer Express Software (Applied Biosystems).

### Gene expression analysis by qRT-PCR

Total RNA was isolated with the RNeasy plant mini kit (Qiagen) and the RNA extraction was performed as described in the manufacturer`s protocol using 100 mg frozen tissue powder (ground with mortar in liquid nitrogen). DNase digestion was used to eliminate genomic DNA contamination. RNA quality was controlled with an Agilent 2100 Bioanalyzer (Aglilent Technologies) and RNA quantity measured on a Nanodrop ND-1000 UV–vis Spectrophotometer (Nanodrop Technologies). 2.5 μg of total RNA was reversed transcribed using SuperScriptVilo cDNA synthesis kit (Invitrogen). For qRT-PCR we used the EXPRESS two-step qRT-PCR universal kit (Invitrogen) with the superscript VILO cDNA synthesis kit (Invitrogen).

Two μl cDNA in a total reaction volume of 20 μl was used for each qRT-PCR reaction. Primers were used at a concentration of 0.5 μM and TaqMan probes at a concentration of 0.2 μM. Final ROX Reference Dye was 0.05 μM. Transcript levels were analysed using a ABI7500 real-time PCR machine (Applied Biosystems) with Fast Cycling Program; 95°C for 20 s and 40 cycles of 95°C for 3 s, and 60°C for 30 s. Glyceraldehyde 3-phosphate dehydrogenase (GAPDH) was used as reference gene. Standard curves were made to control that primer and probe pairs had efficiency close to 100%. *B. distachyon* IRIP genes transcript levels were calculated relative to GADPH gene transcript levels using the comparative threshold cycle method (ΔCt method). Three biological replicates (leaf samples from three different plants) were used to estimate expression levels. Mean and standard deviation of 2^-ΔΔCt^ was calculated for comparison of relative expression levels in CA compared to NA samples. A *t*-test was used to test if ΔCt values of CA samples were significantly different from NA samples (i.e. cold induced gene expression). Three no-template controls for each qRT-PCR plate per gene were performed to control for primer-dimer formation and DNA contamination.

### Expression analysis by microarray

Leaf tissues were ground in liquid nitrogen and total cellular RNA was extracted using RNA Plant reagent (Invitrogen) and RNeasy kits (Qiagen) and treated with RNase-free DNase as previously described [39]. RNA integrity was evaluated using an Agilent Bioanalyzer. Labeled target cDNA was prepared from 125 ng total RNA samples using the NuGen Applause WT-Amp PlusST RNA amplification system Kit protocols (Cat# 5510–24) and Encore Biotin module V2 (Cat# 4200–12). Approximately 4.55 μg fragmented cDNA from each sample was hybridized for 18 hours to an Affymetrix *Brachypodium* Genome Array (BradiAR1b520742). Hybridization was performed using GeneChip® Fluidics Station 450. Arrays were scanned using GeneChip® Scanner 3000 with autoloader at 570 nm and quality-controlled according to the standard Affymetrix protocols (Affymetrix GeneChip® Expression Analysis Technical Manual, 701021 Rev. 5; http://www.affymetrix.com) at the Oregon State University Center for Genome Research and Bioinformatics, Central Service Laboratory (detailed protocols are available at http://www.cgrb.oregonstate.edu/). Image processing and data extraction were performed using AGCC software version 3.0. The Affymetrix eukaryotic hybridization control kit and Poly-A RNA control kit were used to ensure efficiency of hybridization and cDNA amplification. All cDNAs from cold stress treatments and control samples were synthesized at the same time and microarray hybridizations were conducted simultaneously. Each array image was visually screened to discount for signal artifacts, scratches or debris.

Probe level normalization was done with Robust Multi-array Analysis (RMA) utilizing the Affymetrix Power Tools (APT) software package ( http://www.affymetrix.com/partners_programs/programs/developer/tools/powertools.affx; [40]). Probe set summarization and expression estimates for each gene were conducted using the apt-probeset-summarize (1.14.3) program from Affymetrix. Data manipulations were performed using Perl scripts to calculate fold change between normalized treatment and control probe set values.

### Phylogenetic analyses

IRIP, FST, and CBF gene families contain recently derived paralogs (i.e. having few substitutions), hence all phylogenetic analyses were carried out on the nucleotide level. Sets of coding sequences (CDS) of IRIP, FST and CBF3/CBF4 genes from core Pooideae species were assembled from different sources. For the IRIP phylogeny, a representative collection of CDS from both Triticeae and Poeae tribe species were downloaded from NCBI and merged with the identified *B. distachyon* IRIP homologs (see text above). To identify CBF3/4 and FST homologs in *B. distachyon*, the CDS (v1.2) annotation was downloaded from ( http://ftp.brachypodium.org/files/Annotation/) and local blast searches were performed using CBF3/4 genes identified in Skinner et al. [41] as queries. The CBF3/4 phylogeny were constructed using CBF3/4 genes from Triticeae [41], Poaea [42], and rice [30]. Additional barley CDS sequences were collected from a large collection of full length (fl) cDNA and used in one analyses of the FST phylogeny. CDS were predicted from fl-cDNA sequences with orfpredictor ( http://proteomics.ysu.edu/tools/OrfPredictor.html) using homology information from blastx searches against proteins from rice, maize, sorghum, and *B. distachyon* downloaded from ftp.plantbiology.msu.edu, ftp.brachypodium.org, ftp.maizesequence.org, and ftp://ftpmips.helmholtz-muenchen.de/plants/sorghum/, respectively. Only target sequences with a blast evalue <1e^-10^ were included in further analyses. Multiple sequence alignments of IRIPs and CBFs were made with MAFFT [37] using a codon model at the Guidance web server ( http://guidance.tau.ac.il) [43]. One hundred bootstrap replicates were calculated for each guidance alignment and sequences below 0.6 sequence score and 0.8 column score were automatically removed from the alignments. The FST alignment were produced with prankster [44] on amino acid residues which were back translated to codons for phylogenetic analyses. All alignments were manually checked and edited in BioEdit [45].

Maximum Likelihood (ML) phylogenetic reconstruction using the best evolutionary model according to Akaike Information Criterion (AIC) was carried out in Treefinder [46]. The trees from ML phylogenetic reconstructions were checked for consistency with alternative Bayesian phylogenetic reconstruction estimated with MrBayes [47]. The model used in the MrBayes analyses were GTR + G + I (lset nst = 6 rates = invgamma), and simulations were run for 1 × 10^5^ or 1 × 10^6^ generations with tree sampling every 100 or 1000 generations, respectively. Average deviations between the split frequencies were <0.01 in all analyses and a ‘burn in’ which excluded half the sampled trees was used to generate a consensus tree. Phylogenetic trees were visualized in MEGA v5 [48].

### Bacteriophage library construction and screening

DNA of the inbred diploid line Bd3-1 was used to construct a bacteriophage lambda genomic library. Bd3-1 genomic DNA was a kind gift from Dr. David Garvin, University of Minnesota, USA. This library was screened with a rice cDNA probe encoding C-Repeat Binding Factor (CBF)/Dehydration Responsive Element Binding Protein (Os-DREB1A), and a probe encoding Os-DREB1B [49]. DREB1A is an CBF3 subfamily CBF while DREB1B is an CBF4 subfamily CBF. The same clones cross-hybridizing to Os-DREB1A cross hybridized to Os-DREB1B, and no additional Os-DREB1B cross-hybridizing clones were identified. All clones fell into one of two classes based on restriction enzyme patterns. Two representative clones were sequenced and these sequences deposited in GenBank (accessions JQ180470 and JQ180471).

### Fructan measurement

We measured total carbohydrate and fructan content in one *B. distachyon* spring (21–1) and winter type (29–1) and in the core Pooideae species *Lolium perenne L*. (perennial ryegrass) and *Phleum pratense L.* (timothy) before and after cold acclimation. Eight plants of each species were grown in the greenhouse under 16 h photoperiod. After eight weeks, half the plants were placed in a cold chamber at 2°C, while the other half was kept in the greenhouse as control. Leaf tissue from cold treated and control plants was harvested after four days and stored at −80°C. Extractions of total carbohydrate and fructan were carried out as described in Thorsteinsson et al. [50]. For the colorimetric quantification we made slight modifications to the method described in Pollock [51]; because levan is the principal monocot fructan in grasses [52,53] we chose levan as our fructan standard instead of inulin. Different concentrations (0.2, 0.4, 0.6, 0.8, 1, 2, 3, 4, and 5 mg ml^-1^) of levan and glucose were used to make separate standard curves.

Fructan samples were analyzed by high-performance anion-exchange chromatography (HPAEC) on a Dionex ICS3000 system (Dionex Corp.). Two μl of each filtrate was injected on a CarboPac PA1 column (2x250 mm analytical column with a 2x50 mm guard column) operated at 30°C, with 0.25 ml min^-1^ and analyses were detected with pulsed amperometric detection (PAD). Analyte separation was obtained by applying a gradient of eluent A (100 mM NaOH) and B (1.0 M NaOAc in 0.1 M NaOH) starting at 100% eluent A, followed by a two min linear gradient to 5% eluent B, then increasing to 25% eluent B in 23 min and a final increase to 50% eluent B reached at 45 min was kept for three min. Column reconditioning was obtained by returning to initial conditions in one min which was kept for 10 min. The following external standards were used for peak identification; levan (Sigma-Aldrich), fructose (Sigma-Aldrich), glucose (Sigma-Aldrich), sucrose (Sigma-Aldrich), and a set of fructooligosaccharides (1-Kestose, Nystose and 1-Fructofuranosylnystose) (Wako Chemicals).

## Results and discussion

### Cold responsive IRIP genes evolved early in the pooideae lineage

**Figure 2:**
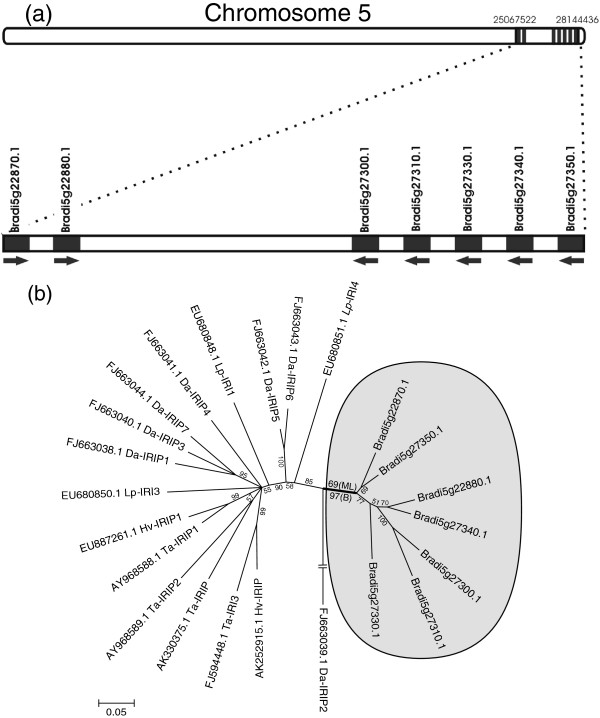
**Localization and distribution of seven IRIP genes identified in *Brachypodium distachyon *on chromosome 5 and *B. distachyon *IRIP genes phylogeny. (a)** The location of seven *Brachypodium distachyon* IRIP genes on chromosome 5. **(b)** 50% consensus ML phylogeny (model J2 + G) of a subset of IRIP sequences from core Pooideae species and all 7 *B. distachyon* IRIP homologs based on 100 bootstrap replicates. The clade support from the alternative Bayesian phylogeny (1.000.000 generations) is shown together with the ML bootstrap support at the split which defines *B. distachyon* IRIP genes as monophyletic. Species abbreviations are as follows: Lp, *Lolium perenne*; Da, *Deschampsia antarctica*; Hv, *Hordeum vulgare*; Ta, *Triticum aestivum.*

**Figure 3:**
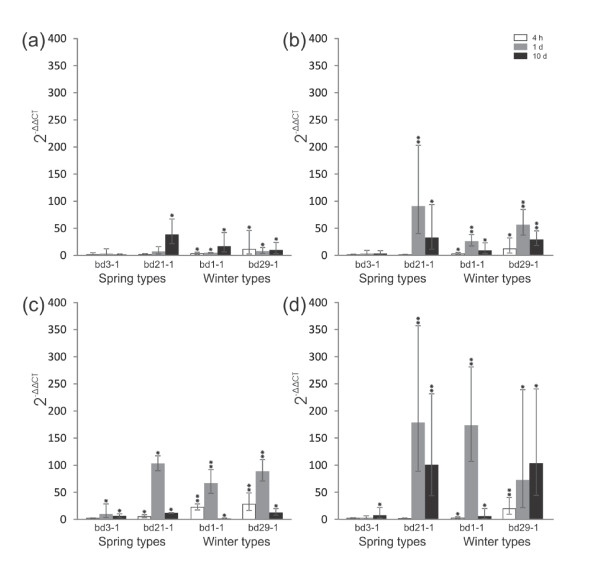
**Quantitative reverse transcript PCR analyses of *Brachypodium distachyon *IRIP genes at three timepoints after cold acclimation. (a)** Bradi5g27300.1 **(b)** Bradi5g27310.1 **(c)** Bradi5g27330.1 **(d)** Bradi5g27350.1 The expression values are given as 2^-ΔΔCt^ and stars above bars denote the significance levels of *t*-test (*P < 0.05, **P < 0.001). Single extreme outliers were removed from four samples before estimation of expression levels and standard deviations (see Additional file 2).

Seven *Brachypodium distachyon* IRIP-like genes from two gene clusters containing two and five genes were identified on chromosome 5 (Figure 2a). Bradi5g22870.1 and Bradi5g22880.1 in the proximal cluster have both truncated ice binding domains (Additional file 1). To test whether the *B. distachyon* IRIPs are induced by low temperature, paralog specific qRT-PCR primers were designed for four IRIP paralogs with non-truncated ice-binding domains (Bradi5g27300.1, Bradi5g27310.1, Bradi5g27330.1, Bradi5g27350.1) (Table 1). Strong cold induction of all genes except Bradi5g27300.1 was observed after one day of cold acclimation in both winter types and the 21–1 line (Figure 3). Bradi5g27300.1 was cold induced in three genotypes, but the level of expression was generally lower than the other IRIP genes (Figure 3, Additional file 2). Expression data from the microarray experiment confirmed the general patterns of IRIP gene cold induction observed in the qRT-PCR experiment; little or no cold induction of Bradi5g27300.1 and medium to strong (2.5-25 fold) cold induction of the other IRIP genes within 24 hours cold treatment (Table 2).

**Table 1:** Primer sequences used for qRT-PCR experiments

**Gene name**	**forward primer 5′ > 3′**	**reverse primer 5′ > 3′**	**probe 5′ > 3′**	**product size (bp)**
Bradi5g27300.1	ggctaccggacaaccaaata	aacgttgttgtccccagtg	ccggggccaacaactctgtca	109
Bradi5g27310.1	aacactgttatgggggagga	ggatacgctattgttgctgcc	tggggacaacaacgttgtgtctgg	120
Bradi5g27330.1	ttcgaaacaggttccttgct	agcacacggaggtcatcg	gcaataagcacggcggtggc	121
Bradi5g27350.1	aaccacaacaaaatcctaagtgg	gttgtggctcctggtcacg	tgccgtaagtggtcacatgcatg	117
BradiGAPDH	ggtgccaagaaggttgtcat	ggtgccaagaaggttgtcat	gcacccagcaaagatgctccc	190

**Table 2:** **Gene expression values from microarray study of cold induction of IRIP, CBF*3*and FST-like homologs in*Brachypodium distachyon***

		**Fold change values (treatment/control) for each time point**				
**Gene family**	**Gene name**	**1 hr**	**2 hr**	**5 hr**	**10 hr**	**24 hr**
IRIP	Bradi5g27350.1	1.31	1.31	1.40	3.84	24.18
	Bradi5g27340.1	1.39	0.86	2.23	6.13	14.38
	Bradi5g27300.1	1.40	0.80	1.10	0.46	0.89
	Bradi5g27310.1	0.92	1.01	0.54	0.91	2.92
	Bradi5g27330.1	1.95	0.87	3.57	23.61	25.46
CBF3c/d	Bradi4g35630.1	105.40	30.19	21.27	1.94	2.48
	Bradi1g57970.1	0.74	0.66	1.42	0.77	1.23
	Bradi4g35570.1	66.85	191.10	262.59	10.89	1.43
	Bradi2g60331.1	20.49	32.94	10.98	7.32	4.61
	Bradi2g60340.1	5.19	5.61	2.46	1.84	1.25
	Bradi3g57360.1	0.62	0.69	0.79	0.87	1.04
	Bradi4g35590.1	3.67	5.66	5.20	2.38	0.71
	Bradi4g35600.1	41.81	59.32	24.30	13.55	2.07
	Bradi4g35610.1	3.82	6.53	3.73	0.76	0.49
	Bradi4g35620.1	5.24	8.56	4.69	4.25	1.51
	Bradi1g77120.1	17.02	11.02	6.87	12.16	2.26
FST-homologs	Bradi3g00910.1	2.44	2.82	4.88	3.35	1.67
	Bradi1g52210.1	1.61	1.04	1.08	0.81	0.61

Expression values given as fold change values (treatment/control).

*Note: gene expression data unavailable for Bradi4g35580 and Bradi4g35640.

The large standard deviations of the qRT-PCR expression values obscures detailed analyses of expression differences, nevertheless one interesting pattern in the IRIP expression data is apparent. In our limited dataset, the winter types express IRIPs more rapid than the spring types. For three of the four IRIP genes only the winter types have significant p-values at 4 hours (Figure 3a-b,d), while the fourth IRIP gene (Figure 3c) has marked higher expression in winter types at 4 hours, compared to Bd21-1. The pattern of low IRIP gene induction early in the CA treatment (<5 hrs) is also observed for Bd21-1 in the microarray experiments (Table 2). One hypothesis is therefore that winter types have a more rapid IRIP gene cold induction compared to spring types. It must be noted that expression data from many more lines of different flowering habits is needed to test this hypothesis.

Occurrence of *bona fide* IRIP genes in *B. distachyon*, containing the conserved ice-binding domain and being cold responsive*,* places the evolution of IRIP’s early in the Pooideae evolution prior to the *Brachypodium*-core Pooideae divergence. Moreover, the phylogenetic analysis supports a monophyletic origin of the *B. distachyon* IRIP genes (Figure 2b) which means that independent IRIP gene family expansions occurred in the *Brachypodium* lineage after the divergence from core Pooideae species.

### Low IRIP induction in Bd3-1 could be explained by extreme spring type habits

The Bd3-1 spring type showed dramatically lower IRIP cold induction compared to the other lines, with only three qRT-PCR measurements being significantly higher than the NA samples (Figure 3). Since the spring type Bd21-1 has strong cold induced IRIP gene expression, the Bd3-1 IRIP expression phenotype cannot be related to the spring type life strategy per se. A recent vernalization response study showed that Bd3-1 is a rapid flowering spring type which expresses the flowering promoting genes VRN1 and VRN3 at very high levels early in the life cycle. In fact non-vernalized seedlings of Bd3-1 had approximately 4- and 6-fold higher expression levels of one of the two VRN1 paralogs and VRN3, respectively, compared to Bd21 [4]. The physiological transition from vegetative to generative growth stage in cereals is associated with VRN1 induction, repression of cold induced gene expression, and loss of freezing tolerance [54]. Thus, the strikingly low IRIP gene expression observed in Bd3-1 could be related to the very rapid transition from vegetative growth form to flowering observed in this line [4].

### *Brachypodium distachyon* has a large cold responsive CBF3 family but lack CBF4 genes

**Figure 4:**
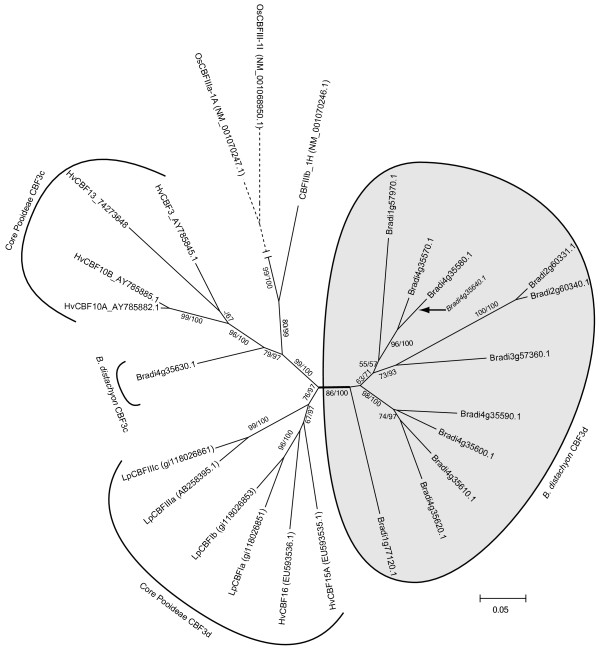
***Brachypodium distachyon *CBF3c/d and core Pooideae CBF3c/d phylogenetic analysis.** Fifty % consensus ML phylogeny (J2 + G model) with 100 bootstrap replicates showing the relationships between the *B. distachyon* CBF3c/d members and core Pooideae sequences. Bradi4g35640 is truncated and were taken out in the final CBF3c/d analyses to increase the number of aligned sites and improve phylogenetic estimations. The placement of Bradi4g35640 is indicated by an arrow. Dotted branches have been scaled down 50%. Species abbreviations are: Hv, *Hordeum vulgare*; Lp, *Lolium perenne*ML bootstrap and Bayesian support values are indicated ML/Bayesian. If identical only one value is shown. ‘-‘ indicates support values below 50.

Fourteen out of the total 18 *B. distachyon* CBF3/4 gene homologs identified in the blast search could be classified as CBF3 members according to the phylogenetic analysis of all CBF homologs (Additional file 3). Thirteen of these genes belong to the CBF3c/d clades. Figure 4 shows the phylogeny of the CBF3c/d genes in *B. distachyon* and two core Pooideae species. One *B. distachyon* gene (Bradi4g35630.1) belongs to the CBF3c subgroup, while the other 12 belong to the CBF3d subgroup. Both the single CBF3c (Bradi4g35630.1) gene member and the majority of the CBF3d genes in *B. distachyon* belong to a gene cluster on chromosome 4. This chromosome is in large parts syntenic with Triticeae chromosome 5 [1] which contains clusters of cold induced wheat and barley CBF3c/d genes [55,56]. Microarray expression data shows that all but two CBF3d genes (Bradi1g57970.1 and Bradi3g57360.1) are cold induced (>2 fold) during 24 hours of cold exposure (Table 2). Together with the phylogenetic analyses this expression data suggests that both cold responsive CBF3c and CBF3d genes were present in a Pooideae ancestor prior to *Brachypodium* and core Pooideae divergence. 

A monophyletic origin of all *B. distachyon* CBF3d paralogs is supported by both ML and Bayesian phylogenetic reconstructions (Figure 4). Moreover, several CBF3d members are found on chromosome 2 and 3 (Figure 4), which does not conform to the syntenic Triticeae 5 relationship [1]. Taken together, this data indicate extensive *B. distachyon* specific duplications of CBF3d genes, both tandem and to other chromosomes, even though alternative scenarios could explain the observed CBF3d topology. First, what appears to be a *B. distachyon*-specific CBF3d clade could have evolved prior to *Brachypodium* divergence and later lost in core Pooideae. Second, gene conversion may homogenize gene sequences and create an ‘illusion’ of evolutionary relatedness [57]. Lastly, all sequence orthologs of the *B. distachyon* CBF3d genes might not yet have been discovered in core Pooideae species.

Surprisingly, none of the 13 *B. distachyon* CBF homologs identified in the blast analyses belonged to the CBF4 group genes (Additional file 3). Screening a bacteriophage lambda genomic library for *B. distachyon* CBF gene content also failed to recover CBF4 homologs from *B. distachyon*. Two phage clones, λBd1C (JQ180470) and λBd5D (JQ180471), harbored four and three CBF*3* genes, respectively, and comparison to the Bd21-1 genome showed that λBd1C and λBd5D correspond to two regions on Bd21-1 chromosome 4. The sequence encompassed by both clones was colinear with the Bd21-1sequence over their entire length. Because rice contains a single CBF4 gene (OsCBF4 AY785894), the most parsimonious model to explain the absence of CBF4 in *B. distachyon* is lineage specific gene loss.

### *Brachypodium distachyon* lacks FST genes and differs from core pooideae species in fructan accumulation during cold stress

**Figure 5:**
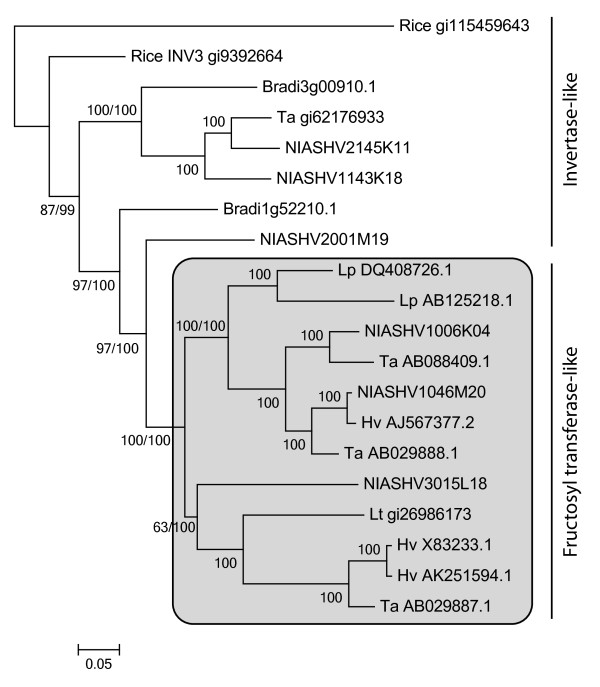
**Phylogeny analysing *Brachypodium distachyon *FST and invertase genes compare to core Pooideae FST and invertase genes.** Fifty % consensus ML phylogeny (GTR + G + I model) of FST genes from core Pooideae species, *B. distachyon* FST homologs and two rice invertase-like genes which are grouped in invertase-like and fructosyl transferase-like based on diagnostic amino acid motifs W(A/S/G)W and WMNDPNG from Lasseur et al. [58]. Species abbreviations are: Os, *Oryza sativa*; Lp, *Lolium perenne*; Lt, *Lolium temulentum*, Hv, *Hordeum vulgare*; Ta, *Triticum aestivum*. Genes named ‘NIASHv’ are from the barley full-length cDNAs database. ML bootstrap and Bayesian support values are indicated ML/Bayesian. If identical only one value is shown. ‘-‘ indicates support values below 50.

In total, fifteen genes with some homology to the core Pooideae FSTs were identified. Thirteen of these were more distantly related than the closest FST homolog in rice (an invertase-like gene), and were thus not considered in the analyses. Core Pooideae FST genes encode a diagnostic h(A/G)Y/F motif [58] and are induced by low temperatures [59,60]. Neither of the two FST-like homologs in *B. distachyon* encoded the FST-motif, but just the invertase motif (Figure 5). The most distant homolog to the FSTs (Bradi3g00910.1) was cold induced in Bd21 (Table 2) and belongs to a monophyletic cluster of invertase-like genes containing gene members from core Pooideae species (Figure 5). Bradi1g52210.1 was placed closest to the FSTs in the phylogeny, but was not cold induced in Bd21-1 within 24 hours of low temperature treatment (Table 2). Some FST-genes are known to be induced by cold only after several days of CA [60], but unfortunately we do not have experimental data for *B. distachyon* cold treatments longer than 24 hrs. Hence it is possible that also Bradi1g52210.1 is induced later in the CA process. 

FST gain probably evolved through a mutation in the substrate binding site of a vacuolar invertase gene [61]. A vacuolar invertase in rice is induced by low temperatures [62] and in this study the expression of a vacuolar invertase homolog in *B. distachyon* (Bradi3g00910.1) is shown to be induced by low temperatures. Hence, it is likely that the ancestral FST gene was responsive to cold prior to the evolution of FST function. The observation that the closest homolog outside the FST clade was a barley gene (NIASHV2001M19) supports an evolutionary model of gain of FST-function after the core Pooideae group divergence from *Brachypodium*. However, a less parsimonious scenario of a FST gene(s) loss in *Brachypodium* could also explain the phylogenetic results and thus cannot be ruled out. Compared to a FST gain in core Pooideae, a ‘loss in *Brachypodium*’ scenario would require a higher number of evolutionary changes (i.e. 2 changes) to be consistent with the topology in Figure 5; in addition to the FST loss, multiple FST gains must have occurred in the Pooideae lineage, or the NIASHV2001M19 barley gene must have reverted back from a FST gene to an invertase-like gene.

**Figure 6:**
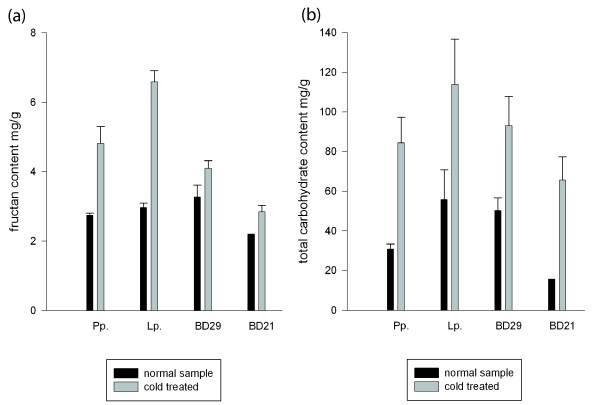
**Carbohydrate accumulation in *Brachypodium distachyon *and core Pooideae species in response to four days of cold acclimation at 2°C.(a)** Fructan contents (mg ml^-1^) and **(b)** total carbohydrate content (mg ml^-1^) from colorimetric quantification. Core Pooideae species are *Lolium perenne* and *Phleum pratense*, while the *B. distachyon* lines used were the spring type Bd21-1 and winter type Bd29-1.

To test whether *B. distachyon* accumulated fructans in response to low temperatures, total carbohydrate and fructan levels were measured before and after cold acclimation. Both core Pooideae species and *B. distachyon* showed marked responses in carbohydrate accumulation during cold stress (Figure 6) as has been shown for many plant species [63-67]. Interestingly, cold induced fructan content increases were much higher in core Pooideae (0.8-1.2 fold) than in *B. distachyon* (0.2-0.3 fold). Qualitative analysis by HPAEC confirmed that both *B. distachyon* and *L. perenne* induce a large increase in fructose, glucose and sucrose during low temperature. However, the profile of short oligosaccharides that accumulated in *B. distachyon* was different from core Pooideae (Additional file 4). For example, significant levels of kestose, nystose and other unidentified oligosaccharides are present after cold acclimation of *L. perenne* but not in cold acclimated *B. distachyon* (Additional file 4a). Moreover, sugars with higher degree of polymerization (DP) are also present to a much larger extent in cold treated core Pooideae compared to *B. distachyon* (Additional file 4b). The correlation of cold induced modifications in fructan content and the phylogenetic clustering of presence/absence of amino acid motifs conferring fructosyltransferase activity support that *B. distachyon* does not possess orthologs of the core Pooideae FST enzymes (Figure 5). 

The distribution of fructan synthesising plants is skewed towards ecosystems characterized by intermittent drought and low temperature stress [68]. Furthermore, both correlative [69-72] and transgenic studies [73] provide compelling evidence for an important role of fructans in drought and cold stress tolerance in core Pooideae grasses. Cold stress associated fructan accumulation was historically assumed to be linked to storage of easily accessible energy reserves as plants prepare for winter [74]. However, results from functional studies have provided insights into a more direct role of fructans in abiotic stress protection, as part of stability enhancing complexes of the cell membrane lipid bi-layers during freezing stress [75-77]. It is thus possible that evolution of FST function, and subsequent increase and diversification of this enzyme family, was important for adaptation to environments with increased abiotic stress levels, such as colder ecosystems, in a core Pooideae ancestor.

## Conclusion

**Figure 7:**
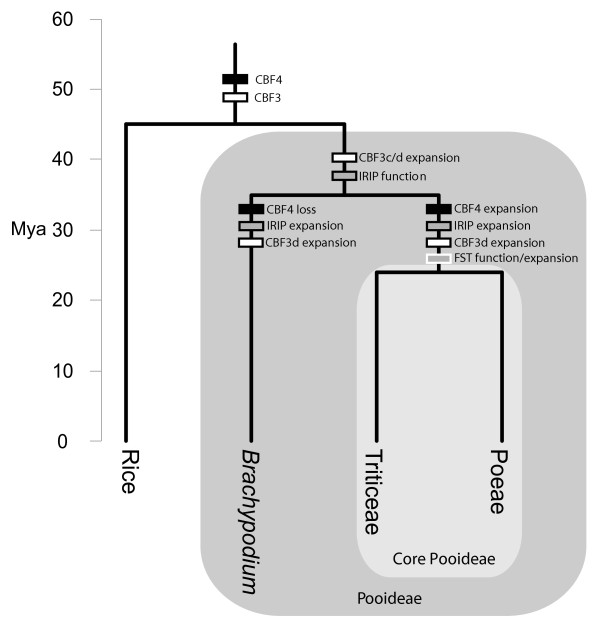
**Summary of the key steps in the evolution CBF3/4, IRIP, and FST genes in Pooideae after the divergence from rice.** The evolutionary steps fall into three categories, ‘function’ (i.e. change of gene function), ‘expansion’ (i.e. gene family expansion), or ‘loss’. Absolute time scale is based on results from [18].

It is evident from our comparative analyses that *B. distachyon* and the core Pooideae differ in key cold stress pathways (Figure 7). Even though this difference limits the use of *B. distachyon* as a holistic model for the molecular biology of low temperature stress in core Pooideae species, *B. distachyon* will be useful to study specific genes and pathways, such as CBF3 or IRIP genes. For example, IRIP RNAi knockout/knockdown lines can be used to test the importance of IRIP gene function for cold and freezing tolerance in Pooideae, and IRIP promoter deletion constructs will be able to shed light on IRIP transcriptional regulation. *B. distachyon* could also be useful to understand the functional divergence between different CBF-gene families, such as the CBF3d and CBF3c group genes, and thus increase our general understanding of transcriptional control of cold stress responses in Pooideae. Since the CBF3c gene is present in single copy in *B. distachyon*, this could facilitate functional analyses and the pathways this gene affect at a mechanistic level.

Differences between *B. distachyon* and core Pooideae also reveal interesting, and potentially biological important, clues to understand the evolution and function of cold acclimation and freezing tolerance in the Poaceae. The absence of genes encoding enzymes in fructan biosynthesis provides a unique opportunity to carry out investigations, using transgenic techniques, to test hypotheses on the evolution of fructan metabolism in relation to adaptation to abiotic stress in grasses.

Understanding the underlying genetic factors controlling climate adaptation within a species is of great importance for breeding of abiotic stress resilience in crop plants. Our study revealed large differences in the transcriptional responses to cold stress among different *B. distachyon* lines. The Bd3-1 spring type had substantially lower levels of IRIP expression at all time-points during cold acclimation (Figure 3), compared to the other Bd-lines in the study. It is possible that this ‘cold non-responsiveness’ is related to the early expression of flowering pathway genes in Bd3-1 [4]. Bd3-1 could therefore be an interesting model to study how mechanistically the transition to flowering (reproductive stage) interact with the CA pathways. Moreover, winter and spring types differed in the transcriptional response time for the IRIP genes. These differences could be related to local adaptations to climatic conditions, and thus provide an interesting model system to study general population differences and adaptation to cold stress responses in Pooideae. Because major transcriptional regulating pathways are conserved across highly divergent species, knowledge about mechanisms for local adaptation in *B. distachyon* populations could be transferable and valuable for agricultural important Pooideae crops.

## Authors’ contributions

CL carried out the experimental work on qRT-PCR gene expression and analyses of total sugar/fructan levels, as well as participating in drafting the manuscript. HR helped design the study, carried out experimental work on gene expression. EJS and HC carried out the screening of *B. distachyon* genomic libraries. EJS also participated in drafting the manuscript. MC was involved in the data acquisition and manuscript preparation. SF collected and analyzed the species distributions and helped draft the manuscript. SEF and TCM carried out microarray based gene expression analysis and participated in drafting the manuscript. BW was responsible for all sugar content analyses and carried out the HPAEC analyses. OAR participated in the design of the study and helped draft the manuscript. SRS participated in the study design, carried out the phylogenetic analyses, and was responsible for the final manuscript preparation.

## Supplementary Material

Additional file 1 **Amino-acid sequences alignment analysis of *Brachypodium distachyon *IRI proteins with *Lolium perenne *IRI proteins sequences.** Sites with with black shade are highly conserved (>70% of sequences). Bradi5g27350.1 and Bradi5g22870.1 have truncated ice binding-domains. Click here for file

Additional file 2 **qRT-PCR expression levels in fold change and p-values of *Brachypodium distachyon *IRIP genes.** Click here for file

Additional file 3 **Minimum evolution CBF3**/**4 gene phylogeny including all *Brachypodium distachyon *homologs.** The Tamura 3-parameter method with gamma distributed rate variation and pairwise deletion was used to calculate evolutionary relatedness. All non-CBF3c/d and CBF4 genes as classified by the phylogeny are in green and red colour, respectively, while other CBF-homologs are in black. Species abbreviations: Os, *Oryza sativa;* Hv, *Hordeum vulgare*; Lp, *Lolium perenne*. Click here for file

Additional file 4 **HPAEC results for carbohydrate contents before and after low temperature treatment.** Detector intensity given as nano coulomb (nC). Black curve and Blue curve are the cold treated and none treated *Lolium perenne* plants carbohydrate extraction elution separately. Purple curve and brown curve are the cold treated and none treated *Brachypodium distachyon* plant carbohydrate extraction elution. (a) Low degree of polymerization oligosaccharides (DP two to five) (glucose, fructose, sucrose, kestose, nystose and other unidentified oligosaccharides) are detected in *L. perenne* and *B. distachyon* using HPAEC. (b) Higher degree of polymerization oligosaccharides are detected in *L. perenne* than in *B. distachyon* using HPAEC. Click here for file
