# Supplementary material for: Comparative analyses reveal potential uses of Brachypodium distachyon as a model for cold stress responses in temperate grasses
Source: BMC Plant Biol. 2012 May 8;12:65. doi: 10.1186/1471-2229-12-65 (PMC3487962; doi:10.1186/1471-2229-12-65)
Supplement: Additional file 1 — Amino-acid sequences alignment analysis of Brachypodium distachyon IRI proteins with Lolium perenne IRI proteins sequences. Sites with with black shade are highly conserved (>70% of sequences). Bradi5g27350.1 and Bradi5g22870.1 have truncated ice binding-domains. [file 1471-2229-12-65-S1.pdf]

```
AY968588_1        1  MAKCGLL-LLFLAFL-LPAA---RATSCHPDDLRALRGFAGNLSGG-AAL-LRAAWSGAS
EU680848_1        1  M---GLL-LLFLGFL-LPAA-CAATSSCHPDDLRALRGFAKNVGGG-GVL-LRTAWSGTS
EU680850_1        1  MAKCLML-LLSFAFL-LSAAGTATATPCHRDDLRALRGFAENLGGG-GALSLRAAWSGAS
EU680851_1        1  MAKCWQL-LLFLALL-LPAA---SAASCHPDDLYALRDFAGNLRGG-GVL-LRAALPGAS
Bradi5g27300      1  MAKCWLLLQLLSALL-LPAA-ISTAAACHPDDLRALRGFAGNLSGG-AVL-LRATWSGAS
Bradi5g27310      1  MAKCWLLMQLLLALL-LPAA-ISMAAACHPHDLHALQGFAGNLSGG-AVL-LRATWTGAS
Bradi5g27330      1  MAKCWLMQLILLTFL-LPAA-ISTAVACHPDDLRVLRGFAGNLSGAGAVL-LRATWSGAS
Bradi5g27340      1  MAKYWLLLQLLSALLILPAA-TSTAASCHPDDLRALRGFAGNLSGGAAVL-LRAAWSGAS
Bradi5g27350      1  MAKCWLP-HLLLALL-LPAA--SMAASCHPDDLRALRGFAGNLSGG-AVL-LRATWSGAS
Bradi5g22870      1  MAKCWLL-HLLLALL-LPAA--STAASCHPDDLHALRGFAGNLSGG-AVL-LRATWFGAS
Bradi5g22880      1  MAKYWLLLQLLSTLLILPAA-TSTAAACHPDDLRALRGFAGNLSGGAAVL-LRAMWSGAS

AY968588_1       54  CCVWEGVNCD-GT-SGRVTALRLPGHGLVGLIPG-ASLAGLARLEELNLANNKL-VG-TI
EU680848_1       53  CCVWEGVGCN-GA-SGRITSLWLPRRGLAGTITG-ASLAGLAGLESLNLANNRL-VG-TI
EU680850_1       58  CCDWEGVGCD-GA-SGRVTALWLPRSGLTGPI----------------------------
EU680851_1       54  CCGWEGVGCD-GA-SGCVKSFQILLKGLTA------------------------------
Bradi5g27300     57  CCGWEGVGCDG--DAGRVTTLSLPERGLAGPIQGAAPLAGLRQLESLNLAHNRLQVGTTF
Bradi5g27310     57  CCGWEGVGCNGGKSDGRVTTLLLPGRGLTGPILGASSLAGLAQLESLNLAHNRLQVGSTF
Bradi5g27330     58  CCSWEGVGCNGGA-SGRVMTLWLPGRGLAGPIQGAASLAGLARLESLNLANNRLQVGTTF
Bradi5g27340     59  CCGWEGVRCDDGA-SGRVTTLWLPGRGLVGPIHGASSLAGLAQLESLNLANNRLHVGTTF
Bradi5g27350     55  CCGWEGVGCD-SA-SGRVTSLWLPGRGLTGPIQGAASLAGLVRLESLNLADNRL-VG-TI
Bradi5g22870     55  CCGWEGVGCD-GA-SGRVTTLWLPGRGLAGPIQGAASLAGLAQLESLNLANNRL-VG-TI
Bradi5g22880     59  CCGWEGVSCDGGA-SGRVTTLWLPGRGLVGPIQGVSSLAGLAQLESLNLANNRLQIGTTF

AY968588_1      109  PSWIGELDHLCYLDLSDNSLVGEVPKSL-IRLKGLVIAGHSLGMVFTNMPLYVK-RNRRT
EU680848_1      108  PSWIGELDHLLYLDLSHNSLVGELPNRLRIRLKGLTTTGHLLGMTFTNMPLDVK-HNRRT
EU680850_1       88  PSWICQLHHLRYLDLSGNALVGEVPKNLQVQLKGI-----------TNMPLHVM-RNRRS
EU680851_1       82  -------------------------------------AGRSLGKAFTHMPLHVK-PSQGT
Bradi5g27300    115  PSWIAEFDRLCYLDLSHNV-----------------------------SPLHVKSSSRRT
Bradi5g27310    117  PLWIGELGHLRYLDLSHNA-----------------------------SPLHVNSSNRRT
Bradi5g27330    117  PSWIGELDRLRYFDLSHNA-----------------------------SSFHVKHGDRRT
Bradi5g27340    118  PSWIGELDRLCYLDLSHNA-----------------------------SPLHVK-RNRRT
Bradi5g27350    111  PSWIGELDRLCYLDLSHNASVYEVAK---------------INPSQRSRGVTVS-TNRKT
Bradi5g22870    111  PSWIGELDRLCYLDLSHNASVDEVHK---------------INPSQRSLGVAVS-TNRRT
Bradi5g22880    118  PSWIGELDRLCYLDLSHNA-----------------------------SPLHVK-RNRRN

AY968588_1      167  LD-EQPNTISGSNNTVRSGSTNVVSGNDNTVISGNNNNVAGSNNTVITGNDNTVTGSNHV
EU680848_1      167  LA-IQPNTISGTNNLVLSGRNNVVSGNDNTVISGNNNTVSGSFNTVVTGSDNILTGSNHV
EU680850_1      136  LD-EQPNTISGSNNTVRSGSKNVLAGNDNTVISGDNNSVSGSNNTVVSGNDNTVTGSNHV
EU680851_1      104  LD-EDHNTITGINNTVRSGSNNVVSGNDNTVISGNNNVVSGSHNTVVFGGDNFISGSYHV
Bradi5g27300    146  LATGQPNTITGANNSVRSGSGNTIMGDGNT-ITGDNNVVSGNN-----------------
Bradi5g27310    148  LAEGQPNTISGTNNSVRSGSGNTVMGEDNIVISGDNNVVSGKQNT-VTGSDNVVSGSN--
Bradi5g27330    148  LADGQPNTITGTNNSVRSGNGNTVSGGDNTVISGNNNVLSGNNNTIISGSDNVVSGTNQV
Bradi5g27340    148  LADGKPNTITGTNNSVKSGNGNTISGNDNVVISGNNKVVCGNHNKVVSGSDNAVSGNMHV
Bradi5g27350    155  LD-EEPNTITGTNNHVRSGKDNALSGNDNTVISGDNNVVTGNHNKLLSGSHNAVSGHMHV
Bradi5g22870    155  LG-GEPNTITGTNNHVRSGKDNALSGSDNTVISGNIAKITTTKS----------------
Bradi5g22880    148  TS----------------------------------------------------------

AY968588_1      226  VSGDKHIVTDNNNAVSGTIMYPGASIP---------------------------------
EU680848_1      226  VSGRSHIVTDNNNSVSGDDNNVSGSFHK-------VSGSHNTVSGSNNT-----------
EU680850_1      195  VSGTNHIVTDNNNNVSGNDNNVSGSFHT-------VSGGHNTVSGSNNT-----------
EU680851_1      163  VSGNHHVVTDNKNAVSGDHNTVSGSQNTVSGNHQIVSGSHSTVSGNHNTVSGRNNSVYGN
Bradi5g27300    188  ----------------------------------------NTVTGSNNT-----------
Bradi5g27310    205  ----------------------------------------NSVSGSHNT-----------
Bradi5g27330    208  VSGTNHIVTGSN----------------------------NTVSGNNNN-----------
Bradi5g27340    208  VSGTHHVVTGTN----------------------------NTVSGSNNA-----------
Bradi5g27350    214  VSGTYHVVTGNNNAVTRSHNTASGNHNIVSGHHNTVSGDHNTVSGSHNT-----------
Bradi5g22870         ------------------------------------------------------------
Bradi5g22880         ------------------------------------------------------------
```

Truncated ice-binding domain
