# Supplementary material for: Comparative analyses reveal potential uses of Brachypodium distachyon as a model for cold stress responses in temperate grasses
Source: BMC Plant Biol. 2012 May 8;12:65. doi: 10.1186/1471-2229-12-65 (PMC3487962; doi:10.1186/1471-2229-12-65)
Supplement: Additional file 2 — qRT-PCR expression levels in fold change and p-values of Brachypodium distachyon IRIP genes. [file 1471-2229-12-65-S2.pdf]

Locus	Cold acclimation	Genotype	Fold change (2-ΔΔCT)	p-values
Bradi5g27300	4hr	bd3-1	1.66	0.28
		bd1-1	3.25	0.01
		bd21-1	1.82	0.18
		bd29-1	11.18	0.01
	1d	bd3-1	2.70	0.26
		bd1-1	4.70	0.01
		bd21-1	6.80	0.02
		bd29-1	7.60	0.01
	10d	bd3-1	1.28	0.32
		bd1-1	16.62	0.01
		bd21-1*	38.26	0.01
		bd29-1	9.32	0.01
Bradi5g27310	4hr	bd3-1	1.33	0.30
		bd1-1	2.84	0.01
		bd21-1	1.27	0.23
		bd29-1	11.94	0.00
	1d	bd3-1	2.85	0.09
		bd1-1	25.70	0.00
		bd21-1	90.25	0.00
		bd29-1	56.17	0.00
	10d	bd3-1	2.75	0.06
		bd1-1	8.55	0.02
		bd21-1	32.28	0.00
		bd29-1	28.77	0.00
Bradi5g27330	4hr	bd3-1	2.20	0.09
		bd1-1	22.38	0.00
		bd21-1*	5.10	0.02
		bd29-1	28.14	0.00
	1d	bd3-1	9.53	0.01
		bd1-1	66.45	0.00
		bd21-1*	102.52	0.00
		bd29-1	88.33	0.00
	10d	bd3-1	5.82	0.01
		bd1-1	1.20	0.34
		bd21-1*	11.58	0.02
		bd29-1	12.38	0.00
Bradi5g27350	4hr	bd3-1	2.16	0.08
		bd1-1	2.54	0.03
		bd21-1	1.78	0.07
		bd29-1	19.84	0.00
	1d	bd3-1	2.40	0.07
		bd1-1	173.40	0.00
		bd21-1	177.97	0.00
		bd29-1	72.18	0.00
	10d	bd3-1	7.19	0.01
		bd1-1	5.31	0.04
		bd21-1	100.34	0.00
		bd29-1	103.19	0.00

*denotes measurement where one extreme outlier has been removed
