# Supplementary figures and images for: Comparative analyses reveal potential uses of Brachypodium distachyon as a model for cold stress responses in temperate grasses
Source: BMC Plant Biol. 2012 May 8;12:65. doi: 10.1186/1471-2229-12-65 (PMC3487962; doi:10.1186/1471-2229-12-65)

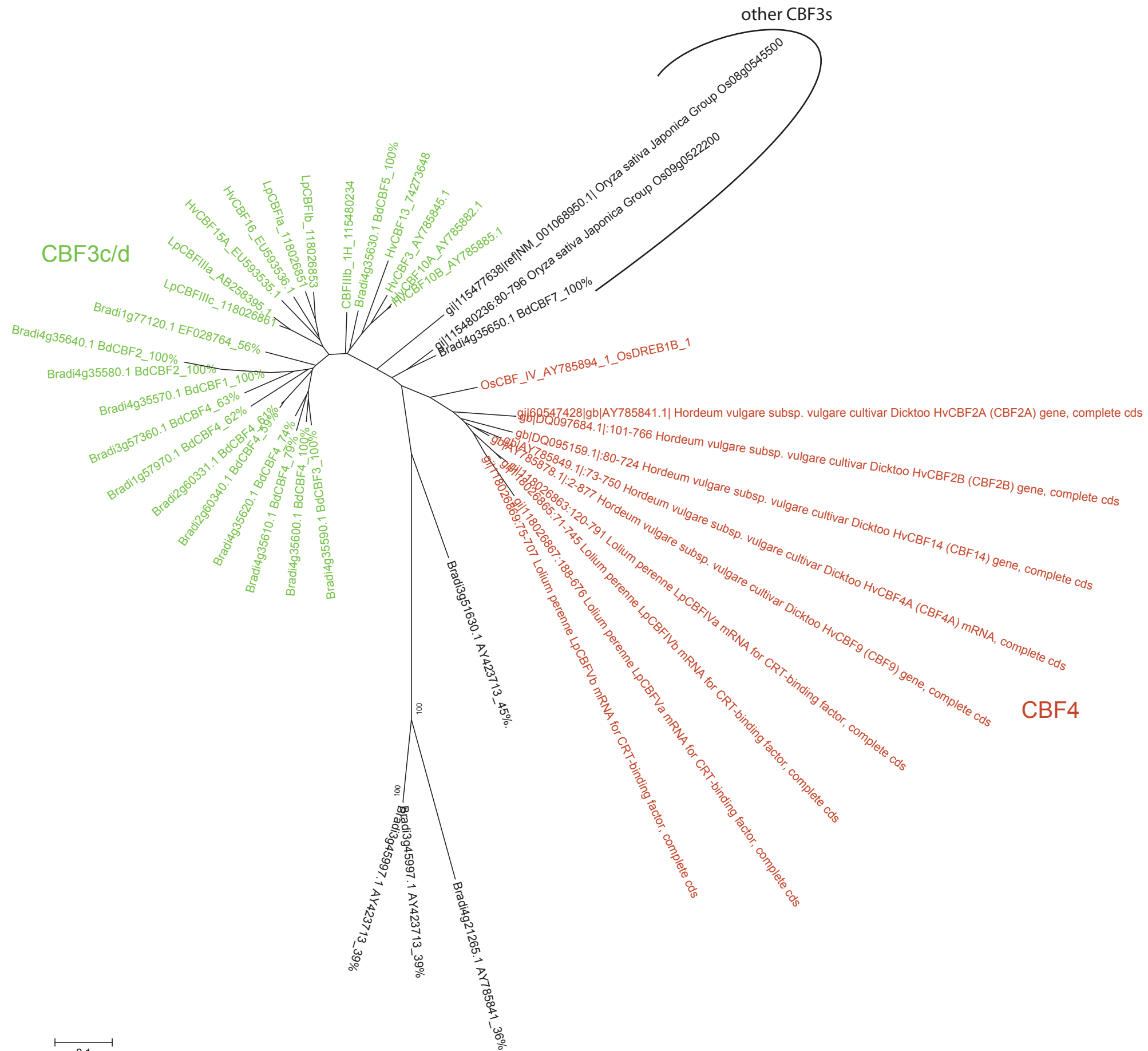

Supplement: Additional file 3 — Minimum evolution CBF3/4 gene phylogeny including all Brachypodium distachyon homologs. The Tamura 3-parameter method with gamma distributed rate variation and pairwise deletion was used to calculate evolutionary relatedness. All non-CBF3c/d and CBF4 genes as classified by the phylogeny are in green and red colour, respectively, while other CBF-homologs are in black. Species abbreviations: Os, Oryza sativa; Hv, Hordeum vulgare; Lp, Lolium perenne. [file 1471-2229-12-65-S3.pdf]

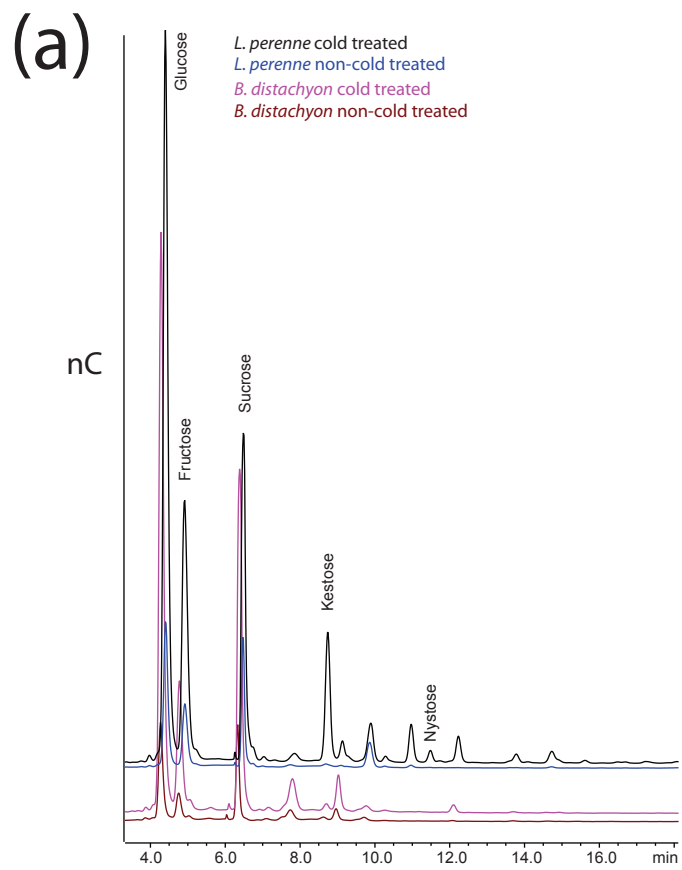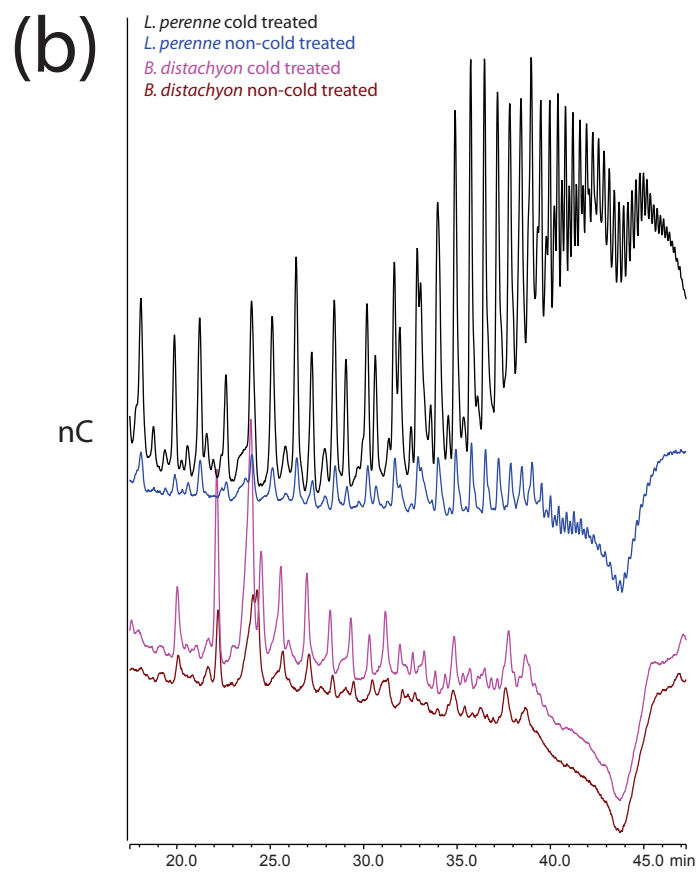

Supplement: Additional file 4 — HPAEC results for carbohydrate contents before and after low temperature treatment. Detector intensity given as nano coulomb (nC). Black curve and Blue curve are the cold treated and none treated Lolium perenne plants carbohydrate extraction elution separately. Purple curve and brown curve are the cold treated and none treated Brachypodium distachyon plant carbohydrate extraction elution. (a) Low degree of polymerization oligosaccharides (DP two to five) (glucose, fructose, sucrose, kestose, nystose and other unidentified oligosaccharides) are detected in L. perenne and B. distachyon using HPAEC. (b) Higher degree of polymerization oligosaccharides are detected in L. perenne than in B. distachyon using HPAEC. [file 1471-2229-12-65-S4.pdf]
